# Aortic Dissection From an Intra-aortic Balloon Pump: A Dangerous Complication to Keep in Mind

**DOI:** 10.7759/cureus.39122

**Published:** 2023-05-17

**Authors:** Ivan A Mijares-Rojas, Luis G Trujillo, Paola A Lecompte-Osorio, Enrique F Martinez Trevino, Mrudula Munagala

**Affiliations:** 1 Internal Medicine, University of Miami Miller School of Medicine, Jackson Memorial Hospital, Miami, USA; 2 Internal Medicine, John H. Stroger, Jr. Hospital of Cook County, Chicago, USA; 3 Cardiology, Miami Transplant Institute, Miami, USA

**Keywords:** intra-aortic balloon pump, aortic dissection management, mechanical circulatory assistant devices, thoracic endovascular aortic repair, thoracic aortic dissection, intra-aortic balloon pump (iabp)

## Abstract

Despite the benefits of the intra-aortic balloon pump (IABP) being a subject of debate, it remains a widely available and easy-to-use mechanical circulatory support device. Nonetheless, its use is not exempt from complications. Aortic dissection from IABP is an infrequent but deathly complication. We describe a case in which early recognition of the condition led to control through an endovascular approach. A 57-year-old male was admitted for acute decompensated heart failure requiring intravenous inotropic agents. While undergoing assessment for a heart transplant, he developed cardiogenic shock requiring initiation of mechanical circulatory support with an IABP. A few hours after device implantation, the patient developed acute tearing chest pain and was found to have an acute dissection in the descending thoracic aorta. Prompt liaison with the endovascular team led to a thoracic endovascular aortic repair to control the extent of the lesion.

## Introduction

Intra-aortic balloon pump (IABP) has been a left ventricle workload reliever for over 50 years. Since its implementation into clinical practice in 1967, patients with a failing heart have benefitted from its hemodynamic and myocardial oxygen demand advantages [1]. Its ease of application, affordability, and accumulative clinician comfort with use have positioned it as the most widely available mechanical circulatory support (MCS) device [2]. Given its presence and usefulness in multiple failing heart scenarios, clinicians must know its common and uncommon complications.

## Case presentation

A 57-year-old male was admitted for acute decompensated heart failure management after presenting with dyspnea and chest discomfort. His medical history included hypertension, hyperlipidemia, chronic kidney disease, peripheral vascular disease, congestive heart failure, and coronary artery disease requiring coronary artery bypass graft (CABG) and percutaneous coronary intervention (PCI) in the past. The last echocardiogram demonstrated severely reduced left ventricular function with an ejection fraction of 10-15%. The patient had already required an implantable internal cardioverter defibrillator for sudden death prevention. Given the development of acute kidney and liver injury on presentation, the patient was admitted to the cardiac critical care unit under hemodynamic support with dobutamine and norepinephrine.

**Figure 1:**
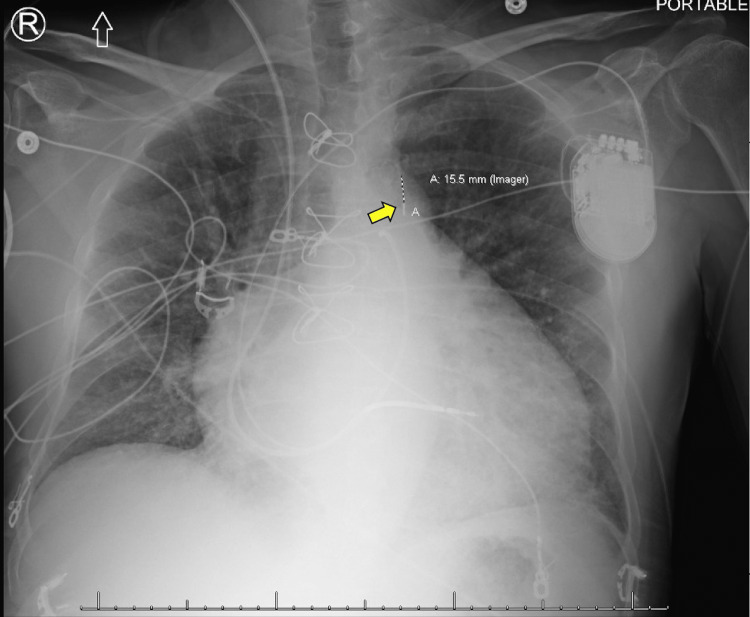
Chest X-ray (anteroposterior view) demonstrating the intra-aortic balloon pump catheter tip (yellow arrow) positioned approximately 1.6 cm from the aortic knuckle.

In a four-day timeframe, the patient was stabilized and tapered down on inotropic agents. He continued to be hemodynamically dependent on low-dose dobutamine infusion (5 mcg/kg/min) and was transferred to the medical floor for a heart transplant candidacy evaluation. Three days later, the patient developed lower extremity edema, concomitant dyspnea, and elevated serum creatinine. A decision was made for hemodynamic assessment in the catheterization lab. Swan-Ganz catheter demonstrated worsening cardiogenic shock with a cardiac index reading of 1.3 L/min/m^2^, and the decision was made for IABP placement. A 40-cm balloon was advanced through the right femoral artery under fluoroscopic guidance and normal function with 1:1 augmentation on systole was recorded. After device implantation, the mean arterial pressure improved from 59 mmHg to 82 mmHg. There were no acute complications, and balloon pump positioning was appropriate under angiography. Chest X-ray demonstrated the tip of the balloon pump at 1.6 cm from the aortic knuckle (Figure 1). Subsequent repositioning to a 2-3 cm mark from the aortic knuckle was performed before leaving the catheterization laboratory with device function interruption for positioning.

**Figure 2:**
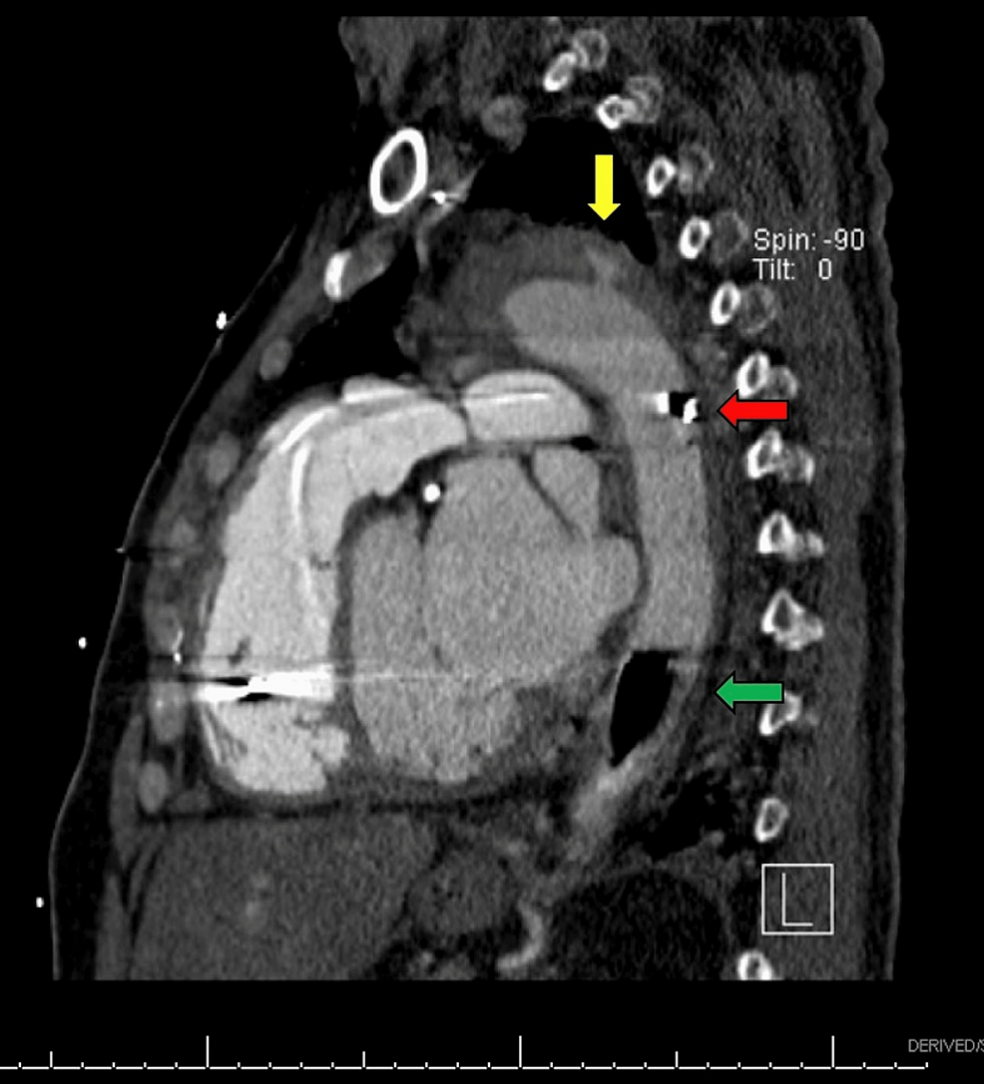
Parasagittal view of CT angiogram demonstrating a tear within the descending aorta with hematoma formation (yellow arrow). Intra-aortic balloon pump catheter tip (red arrow) and balloon (green arrow).

**Figure 3:**
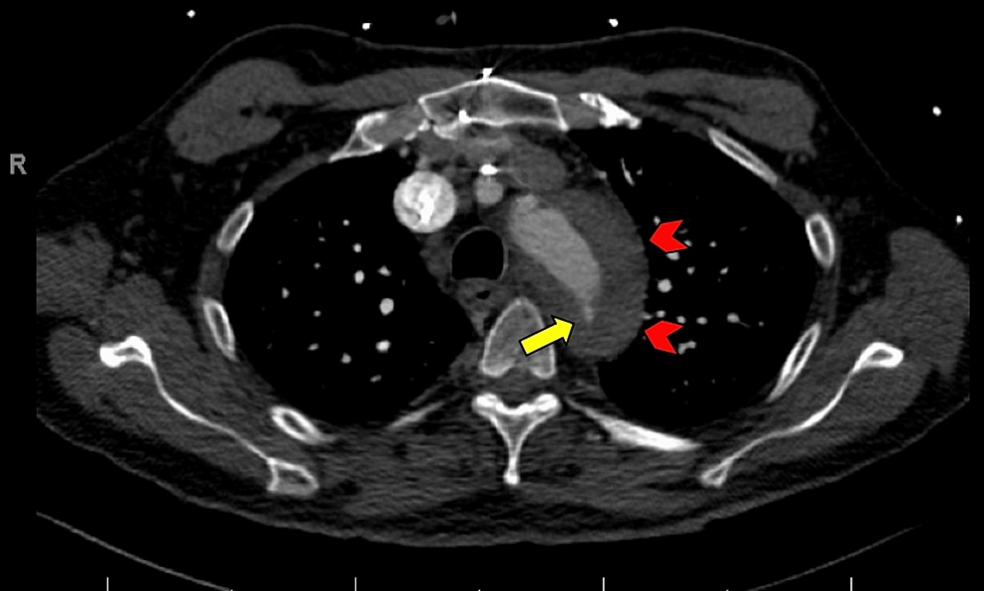
CT (axial view) demonstrating the aortic tear (yellow arrow) and surrounding hematoma formation (red arrowheads).

Approximately 15 hours after the procedure, the patient complained of severe tearing chest pain that radiated to the back in between the scapulae. The pain was not pleuritic in characteristic and was not positional-dependent. The patient remained hemodynamically stable, and IABP functionality was maintained. The electrocardiogram (ECG) did not show ischemic changes, serum troponin level was negative, and a bedside echocardiographic assessment was unchanged compared to baseline. Given the recent IABP insertion, a CT aortic angiogram was considered to rule out aortic dissection. Findings were consistent with an entry tear and intramural hematoma below the origin of the subclavian artery in keeping with acute type B aortic dissection (Figures 2, 3).

**Figure 4:**
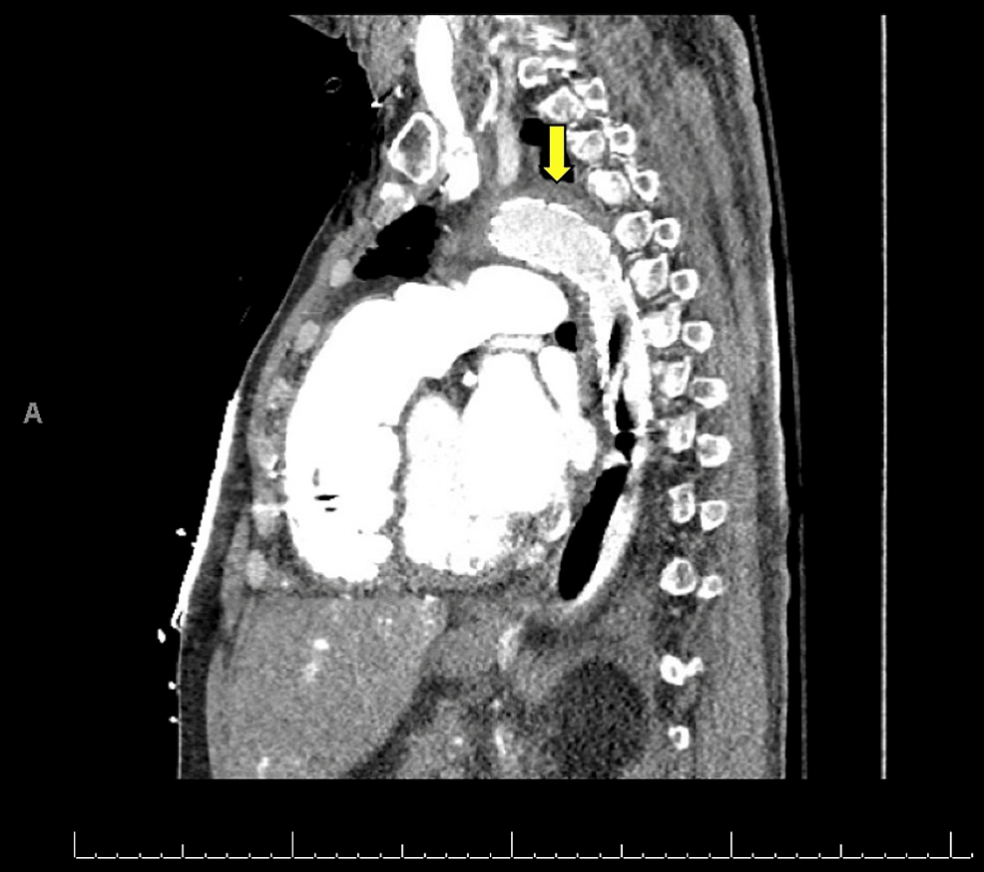
CT angiogram (parasagittal view) demonstrating the presence of a stent graft covering the lesion area at the descending aorta (yellow arrow) and extending distally to cover the intra-aortic balloon pump device.

**Figure 5:**
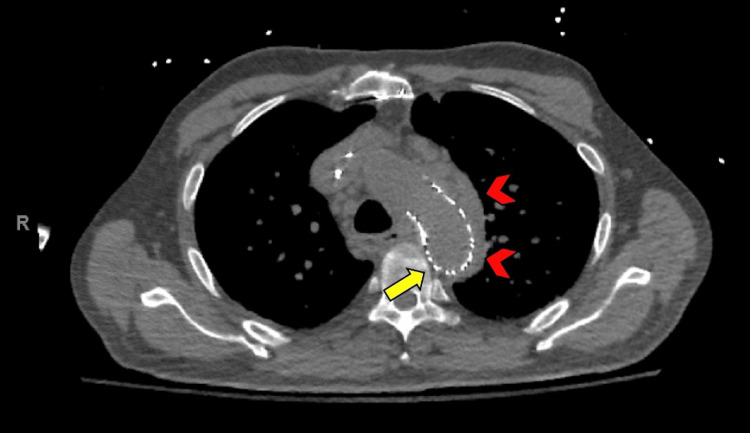
Axial CT cut demonstrating the covering of the lesion with the stent graft (yellow arrow), preventing hematoma enlargement (red arrowheads).

The endovascular team was consulted with a view to thoracic endovascular aortic repair (TEVAR) to address the acute event and prevent further lesion progression. Access was obtained under ultrasound guidance through the left common femoral artery, and angiography plus intravascular ultrasound (IVUS) was performed for aortic lumen measurement. A 31 mm by 15 cm Gore TAG® (W. L. Gore & Associates Inc., Newark, DE, USA) conformable thoracic stent graft was positioned, covering the defect and extending distally to the descending aorta. The long graft was purposely selected to protect the IABP dependency. The procedure had no complications, and the IABP device kept functioning except for downward repositioning on stent-graft deployment. Repeat imaging did not demonstrate an extension of the defect (Figures 4, 5).

IABP was removed on day one following the TEVAR procedure. Subsequently, the patient was placed on biventricular Impella® (Ambiomed Inc., Danvers, MA, USA) device support. Regrettably, he declined further aggressive treatment as a bridge to a heart transplant and died two days later due to cardiogenic shock and multi-organ failure.

## Discussion

Cardiogenic shock (CS) is a high-mortality complex disorder characterized by a severe impairment of myocardial contractility and reduced cardiac output. It can result in life-threatening end-organ hypoperfusion and hypoxia. CS presents with hypotension refractory to volume resuscitation and may require inotropic support, vasopressors, and eventually MCS [3].

The IABP is considered the simplest modality of MCS. While it may offer more modest hemodynamic support than Impella or extracorporeal membrane oxygenation (ECMO), its ease of use, safety profile, and wide availability make it the first-line MCS modality [4,5]. The IABP consists of a catheter with a 25-50 ml balloon on its distal end attached to a console that regulates inflation and deflation. The catheter is most commonly inserted via the femoral or axillary arteries. It is then advanced to its optimal position, with its distal tip located 2-3 cm distal to the origin of the left subclavian artery [6,7].

IABP provides hemodynamic support following the appropriate timing of inflation and deflation with the cardiac cycle. Balloon inflation should occur during diastole, in synchrony with the closure of the aortic valve, increasing diastolic aortic pressure and, therefore, both systemic and coronary blood flow. Meanwhile, deflation should occur toward the end of diastole during isovolumetric contraction and immediately before systole, resulting in reduced afterload and improved cardiac output [8,9].

In the last two decades, the indications for IABP have diminished because of controversial results on its mortality benefits. The most recent American College of Cardiology (ACC) and American Heart Association (AHA) guidelines only have a grade 2A evidence recommendation for patients who develop refractory CS following ST and non-ST myocardial infarction [1]. Nevertheless, current practice takes advantage of this MCS for more clinical scenarios, such as patients with acute decompensated heart failure with hypotension, as an adjunct therapy for high-risk PCI, myocardial infarction with CS due to mechanical complications, low cardiac output state post-CABG, and as a bridge therapy for patients with intractable angina, myocardial ischemia, refractory heart failure, or intractable ventricular arrhythmias [10]. The most recent evidence suggests IABP might benefit left ventricle distention due to veno-arterial ECMO or as a bridge therapy for transplant or left ventricle-assisted device (LVAD) implantation [1]. Conversely, contraindications include the presence of an aortic aneurysm or dissection, uncontrolled bleeding diathesis, uncontrolled sepsis, severe aortic insufficiency, and severe peripheral arterial disease [10].

Although IABP is considered to have a better safety profile among all the modalities of MCS, it is not devoid of possible complications. The Benchmark Registry was the first study to assess an extensive registry of patients who underwent IABP placement, reporting a 2.6% rate of major complications. Major complications were classified as bleeding complications, vascular complications and limb ischemia, infection, stroke, IABP-related mortality, and malfunction of the IABP. Risk factors for major complications have been identified, including female gender, peripheral arterial disease, small body surface area (<1.65 m^2^), and age ≥ 75 years. Additional risk factors for ischemic vascular complications are the duration of IABP support, larger catheter size, diabetes, and a cardiac index <2.2 L/min/m^2^ [11-13]. One study on the complications of IABP placement in patients undergoing cardiac surgical procedures found an incidence of 0.4% of aortic dissections and 0.8% of pseudoaneurysms [14]. Aortic perforation and dissection are rare complications of IABP placement, with only a few reported cases in the literature [15-18].

We report a case of a severe vascular complication in the setting of IABP placement, where the patient developed a small aortic tear with intramural hematoma in the descending aortic arch, suspicious for aortic dissection. Aortic perforation and dissection are rare complications of IABP placement, with only a few reported cases in the literature. In our case, it is unclear what precipitated the development of the lesion. The patient had a high-risk profile for atherosclerotic disease and a long history of hypertension. It is possible that the hemodynamic modifications provided by IABP uncovered an ongoing lesion. On the other side, the IABP catheter tip was measured to be approximately 1.6 cm distal from the aortic knob on the chest X-ray. Although this is not an optimal positioning, angiography did not initially reveal complications in the aortic wall on IABP placement, and repositioning was performed afterward. Older studies have commented on the possibility of developing dissecting aneurysms without intimal injury due to lateral and shearing forces of balloon inflation on the aortic wall. Nevertheless, the determination of all the possible implicated mechanisms is limited, given the low incidence of this complication. It would be more important to emphasize that, in the case presented, early recognition of the condition led to a safe endovascular approach, even in the setting of IABP hemodynamic dependency.

## Conclusions

IABP as MCS remains a widespread and growing used modality with an acceptable safety profile. However, its use is not exempt from risks. Clinicians should remain vigilant for the possibility of aortic injury secondary to the insertion of IABP and the consequent development of pseudoaneurysms, tears, intramural hematomas, and even aortic rupture. In the case presented, an endovascular approach with TEVAR, even with ongoing IABP functionality, effectively controlled the extension of the developing dissection and prevented further hematoma formation.
